# The complete chloroplast genome of *Sophora davidii* (Fabaceae) and its phylogenetic implications

**DOI:** 10.1080/23802359.2021.1999187

**Published:** 2022-03-17

**Authors:** Huigai Sun, Xiaowei Han, Donglai Ma, Yang Li, Tian Kang, Ruipeng Sun, Yuguang Zheng, Junna Song

**Affiliations:** a Traditional Chinese Medicine Processing Technology Innovation Center of Hebei Province, Hebei University of Chinese Medicine, Shijiazhuang, China; b People’s Hospital of Shijiazhuang City, Shijiazhuang, China

**Keywords:** Phylogeny, chloroplast genome, *Sophora davidii*, Fabaceae

## Abstract

*Sophora davidii* (Franch.) Pavol. is a deciduous or evergreen shrubs belonging to the genus *Sophora,* Fabaceae. The roots of *S. davidii* have been traditionally used as a medicinal herb in China to clear internal heat, relieve sore throat, and reduce swelling. Here we sequenced the whole genome of the chloroplast of *S. davidii*. The complete length of the chloroplast genome in *S. davidii* is 153,584 bp, containing a large single-copy region of 83,930 bp, a small single-copy region of 15,008 bp, and a pair of inverted repeats regions of 25,823 bp. The total guanine-cytosine (GC) percentage of the chloroplast genome was 36.7%. A total of 131 genes were annotated from the chloroplast genome of *S. davidii*, including 85 protein-coding genes, 8 rRNA genes and 38 tRNA genes. The phylogenetic analysis showed that *S. davidii* is closely related to the other three species of genus *Sophora*.

*Sophora davidii* (Franch.) Pavol., also called David's sophora or *Sophora viciifolia*, is a deciduous or evergreen shrubs belonging to the genus *Sophora,* Fabaceae. *S*. *davidii* is mainly distributed in hill slopes and sandy places in valleys in Southwest China, including Guangxi, Guizhou, Hunan, Sichuan, and Yunnan provinces of China (Tai et al. 2011). The roots of *S. davidii* have been traditionally used as a folk medicinal herb in China to clear internal heat, relieve sore throat, and reduce swelling (Zhao 2004). The flavonoid-rich extract of *S. davidii* showed a good effect in promoting glucose transporter 4 (GLUT-4) translocation and improving glucose uptake in L6 cells (Huang et al. 2018), and several compounds extracted from *S. davidii* promoted GLUT-4 translocations and showed the potential of developing into new anti-diabetic drugs (Li et al. 2021). *Sophora davidii* is often grow on eroded slopes, showing a high tolerance to drought conditions and plays an important role in retaining ecological stability in semi-arid and arid regions (Wu et al. 2008). In addition, *S. davidii* is also cultivated as an ornamental tree.

Chloroplast genome is very conservative in the organizational structure and gene content, consequently chloroplast genome sequences have been widely used in plant evolution and phylogenetics research (Jansen et al. 2007). In the present study, the chloroplast genome of *S. davidii* was sequenced and assembled, and its phylogenetic position was analyzed. The leaves of *S. davidii* were collected from Changdu County, Tibet Autonomous Region, China (31°08′34.84″N, 97°10′17.99″E) and deposited in the Herbarium of School of pharmacy, Hebei University of Chinese Medicine, under the voucher number 20190823-05. Genomic DNA was extracted from the leaves of *S. davidii* with Plant Genomic DNA Kits (Tiangen Biotech Co., Beijing, China). Illumina HiseqX TEN platform was used for genomic DNA sequencing. A total of 10 GB clean reads (paired-end 150 bp) were generated and used for chloroplast genome assembly with NOVOPlasty v 3.7.2. (Dierckxsens et al. 2017). The assembled chloroplast genome was annotated by PGA (Qu et al. 2019). The *S. davidii* chloroplast genome sequence was submitted and deposited in GenBank under the Accession number MN841456.

The complete chloroplast genome of *S. davidii* is 153,584 bp in length, with a classical quadripartite structure, including a large single-copy region of 83,930 bp, a small single-copy region of 15,008 bp, and a pair of inverted repeats regions of 25,823 bp. The total guanine-cytosine (GC) percentage of the genome was 36.7%. A total of 131 genes were annotated in the chloroplast genome of *S. davidii*, including 85 protein-coding genes, 38 tRNA genes, and 8 rRNA genes. There are two copies of *rpl*2, *rps*23, *trn*I-CAU, *ycf*2, *trn*L-CAA, *ndh*B, *rps*7, *ycf*15, *trn*V-GAC, *rrn*16, *trn*I-GAU, *trn*A-UGC, *rrn*23, *rrn*4.5, *trn*R-ACG, and *trn*N-GUU in the chloroplast genome of *S. davidii*. *rps*19 is located at the boundary of LSC/IRB, *ycf*1 gene is located at the boundary of SSC/IRA, *ndh*F gene is located at the boundary of IRB/SSC, and *trn*H-GUG is located at the boundary of IRA/SSC.

To reveal the evolutionary status of *S. davidii*, maximum likelihood method was used to analyze the phylogenetic status of *S. davidii* and other 11 related plant species. The chloroplast genome of *Robinia pseudoacacia* was selected as the outgroup. The phylogenetic tree was reconstructed by using MAGE-X (Kumar et al. 2018). The phylogenetic tree showed that the *S. davidii* and the other three species of genus *Sophora*, i.e. *S. alopecuroides*, *S. flavescens*, *S. tonkinensis* were closely clustered into a clade, and this clade was clustered into a big clade with other four plant species in Sophoreae (Figure 1). The chloroplast genome of *S. davidii* provides important data for the further study of the genetic diversity and conservation biology of species of Sophoreae, Fabaceae.

**Figure 1.**
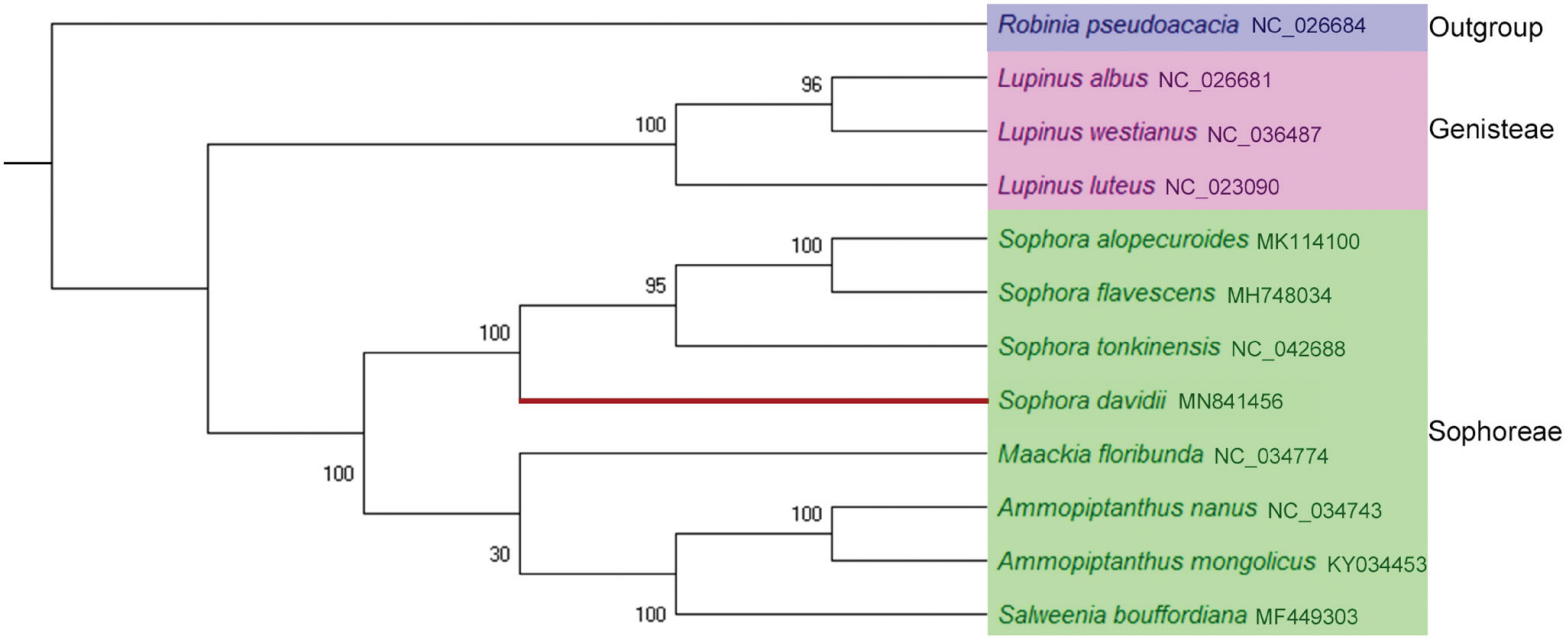
The phylogenetic tree of *Sophora davidii* and the other 11 plant species. The outgroup was *Robinia pseudoacacia*. The numbers on the branches represent the confidence between the two species or for the clade.

## Data Availability

The genome sequence data that support the findings of this study are openly available in GenBank of NCBI at (https://www.ncbi.nlm.nih.gov/) under the accession no. MN841456. The associated BioProject, SRA, and Bio-Sample numbers are PRJNA744118, SAMN20079504, and SRR15049108, respectively.
